# Abdominal Wall Desmoid during Pregnancy: Diagnostic Challenges

**DOI:** 10.1155/2013/350894

**Published:** 2013-01-03

**Authors:** Johnny Awwad, Nadine Hammoud, Chantal Farra, Farah Fares, George Abi Saad, Ghina Ghazeeri

**Affiliations:** ^1^Department of Obstetrics and Gynecology, American University of Beirut Medical Center, P.O. Box 113-6044, Beirut, Lebanon; ^2^Department of Pathology and Laboratory Medicine, American University of Beirut Medical Center, P.O. Box 113-6044, Beirut, Lebanon; ^3^Department of Surgery, American University of Beirut Medical Center, P.O. Box 113-6044, Beirut, Lebanon

## Abstract

*Background*. Desmoids are benign tumors, with local invasive features and no metastatic potential, which have rarely been described to be pregnancy associated. *Case*. We described the rapid growth of an anterior abdominal wall mass in a 40-year-old pregnant woman. Due to its close proximity to the enlarged uterus, it was misdiagnosed to be a uterine leiomyoma by ultrasound examination. Final tissue diagnosis and radical resection were done at the time of abdominal delivery. *Conclusion*. Due to the diagnostic limitations of imaging techniques, desmoids should always be considered when the following manifestations are observed in combination: progressive growth of a solitary abdominal wall mass during pregnancy and well-delineated smooth tumor margins demonstrated by imaging techniques. This case emphasizes the importance of entertaining uncommon medical conditions in the differential diagnosis of seemingly common clinical manifestations.

## 1. Introduction

Desmoid tumors are benign slowly growing fibroblastic neoplasms that arise from muscle fascia or aponeurosis. Although they lack metastatic potential, they are known for their propensity for local recurrence, even after complete surgical resection. Despite being histologically benign, they are locally infiltrative and can cause death through destruction of adjacent vital structures and organs. These rare tumors occur in patients with familial adenomatous polyposis and in patients with previous surgical trauma (previous surgery). Although uncommon, desmoids are found to be associated with women of fertile age, especially during and after pregnancy [1–6].

In this paper, we report on a desmoid tumor that developed during the course of pregnancy and was misdiagnosed to be a uterine leiomyoma by ultrasonography. Final diagnosis and treatment were made at the time of cesarean delivery.

## 2. Case Presentation

A 40-year-old woman presented for cesarean delivery for fetal breech presentation at 39 weeks' gestation under spinal anesthesia. Her previous obstetrical history included two previous first trimester abortions and a full-term pregnancy delivered by cesarean section due to breech presentation. Her present antenatal course was smooth, except for a progressive painless swelling in the right flank noticed at 20 weeks' gestation. Multiple ultrasound examinations were done at different intervals during the course of pregnancy. They demonstrated a rapidly growing sharply defined abdominal mass, heterogeneous in echogenicity and smooth in contour, highly suggestive of a subserosal uterine leiomyoma. No other signs and/or symptoms were reported. The patient denied smoking, drinking alcohol, or taking any medications besides her prenatal vitamins.

**Figure 1:**
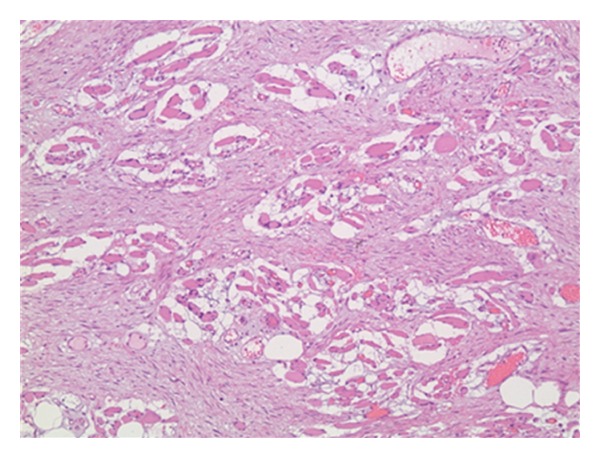
Desmoid tumor spindle cells surrounding and destroying skeletal muscle cells (hematoxylin-eosin, original magnification ×100).

Intraoperatively, the tumor was found to be extra-peritoneal originating from the right abdominal wall. It was whitish in color, hard in consistency, and fixed to its surroundings. It was arising from the lateral wall (rectus and external oblique muscles) reaching the vicinity of the anterior superior iliac spine. Following the delivery of a live male newborn (Birthweight: 3560 g; Apgar score 9 at 5 min) through a lower segment transverse uterine incision, a general surgeon was called in for an intraoperative consultation. Frozen sections revealed benign spindle cells suggestive of a desmoid tumor (Figure 1), although a sarcoma could not be ruled out in view of the tumor rapid growth. The patient consented to a wide surgical excision of the mass within the same surgery. Radical resection of the tumor was performed including affected abdominal wall muscle and a peripheral margin of healthy tissues surrounding it. The patient tolerated the procedure well. She developed right femoral lateral cutaneous neuralgia that resolved spontaneously a few weeks later.

Grossly, the tumor was a 12 × 9.5 × 7 cm mass weighing 457 g, ovoid in shape, firm in consistency, covered by a smooth glistening layer with some attached adipose tissue in areas. Cut sections showed tan fleshy softer tissue in the inside. This was surrounded by a capsule and an inner rim of brown gray firm tissue measuring 0.6 cm in average thickness. The final diagnosis of musculoaponeurotic fibromatosis/desmoid tumor was made.

Two years later, the same women gave birth to her third child by cesarean section. Surgical inspection of the previous resection site was unremarkable. There was no evidence of tumor recurrence.

## 3. Discussion

This case report demonstrates that abdominal wall tumors detected after the first trimester of pregnancy may be clinically challenging and may be easily confused with subserosal uterine leiomyomata. Ultrasonographic features are all too often nonspecific in suggesting a definitive diagnosis. An abdominal wall solid mass exceeding 5 cm in diameter may be difficult to separate by imaging techniques from an enlarged second- or third-trimester gravid uterus. This case emphasizes the importance of entertaining uncommon medical conditions in the differential diagnosis of seemingly common clinical manifestations.

Desmoid tumors are cytologically bland fibrous neoplasms of musculo-aponeurotic origin. Their incidence in the general population is relatively rare where it is detected in only two to four per million individuals each year [2].

Although the etiology of desmoid tumors remains unknown, increasing evidence points to involvement of the APC gene and beta-catenin in the molecular pathogenesis of inherited desmoids (Gardner's syndrome) as well as in sporadic cases [1, 2, 5]. Since desmoid tumors have been frequently associated with high estrogen conditions, an endocrine etiology has been suggested. First, occurrence of extra-abdominal and abdominal desmoids has been reported in women during or after pregnancy [2, 3]. Second, the fibroblast has been shown to exhibit a proliferative response to estrogen. Finally, women with desmoids had regression of their lesions after attaining menopause [1].

The typical clinical manifestation of desmoids consists of a slowly growing deep-seated painless or minimally painful mass. The most common location of desmoids occurring during pregnancy is within the abdominal wall [2]. Their classical presentation is that of an enlarging abdominal mass separate from the uterus. Trauma related to pregnancy including a scar from a prior cesarean section [7] and exposure to elevated hormone levels may both be contributory factors [1, 2]. Subsequent pregnancy is not necessarily a risk factor for recurrence or development of new disease in a woman with a pregnancy-related desmoid, although the data are quite limited (three cases described in which there was no recurrence with subsequent pregnancy [2, 8]). Our case constitutes the fourth reported in which no recurrence was detected in subsequent pregnancies.

Ultrasound remains the most frequently utilized imaging modality for initial assessment of masses compatible with a desmoid tumor. Cross-sectional imaging of the affected area with CT or MRI technology may be more helpful in defining the relationship of the tumor to adjacent structures in order to delineate surgical borders and determine surgical respectability [1–4]. There are no imaging characteristics that can reliably distinguish desmoids from malignant soft tissue tumors. Radiologic imaging may nevertheless be misleading particularly when diagnosis is attempted on the second or third trimester of pregnancy at a time when the gravid uterus comes in tight proximity with the abdominal wall, making the delineation of cleavage planes by imaging techniques very challenging. In our case, ultrasound findings did not raise suspicions of an abdominal wall tumor to warrant the use of further diagnostic modalities. An enlarging uterine fibroid remained the working diagnosis until the time of abdominal delivery.

For asymptomatic desmoids, close observation is acceptable strategy [6]. Surgical treatment is reserved for symptomatic manifestations of the tumor and for cosmetic considerations. Complete surgical resection of the tumor with negative microscopic margins is the gold surgical standard of care [1–3]. Radiation therapy is used in patients with radiosensitive desmoid tumors not amenable to surgical resection, local recurrences, or incompletely excised lesions. Chemotherapy and endocrine therapy have also been used to treat desmoid tumors in patients in whom resection is technically impossible because of a widespread tumor infiltration [1, 3, 4].

Although a very rare entity, the diagnosis of desmoid tumors should always be entertained when the following clinical manifestations are observed in combination: mass located to the abdominal wall, progressive pattern of growth during the course of pregnancy, and well-defined smooth tumor margins demonstrated by ultrasound examination. Definitive treatment is wide surgical resection and may be deferred till after vaginal birth or during the course of a cesarean delivery. This case emphasizes the importance of entertaining uncommon medical conditions in the differential diagnosis of seemingly common clinical manifestations.
